# 1*H*-Benzotriazol-1-yl 4-{(*E*)-[4-(dimethyl­amino)­phen­yl]diazen­yl}benzoate

**DOI:** 10.1107/S1600536813000846

**Published:** 2013-01-19

**Authors:** Abdelkrim El-Ghayoury, Leokadiya Zorina, Mostafa Khouili

**Affiliations:** a LUNAM Université, Université d’Angers, CNRS UMR 6200, Laboratoire MOLTECH-Anjou, CNRS-UMR 6200, 2 bd. Lavoisier, 49045 Angers, France; b Institute of Solid State Physics, RAS, 142432 Chernogolovka MD, Russian Federation; c Laboratoire de Chimie Organique et Analytique, Université Sultan Moulay Slimane, Faculté des Sciences et Techniques, BP 523, 23000 Beni-Mellal, Morocco

## Abstract

The title compound, C_21_H_18_N_6_O_2_, was obtained as a by-product of a reaction between (*E*)-4-(4-dimethyl­amino­phenyl­azo)benzoic acid and 2-amino-4-(2-pyrid­yl)-6-(6-pyrid­yl)-1,3,5-triazine, which has a very low solubility, under peptidic coupling conditions, using THF as solvent. The condensation reaction occurred between 1-hy­droxy­benzotriazole and (*E*)-4-(4-dimethyl­amino­phenyl­azo)benzoic acid. The dihedral angle between the benzene rings in the (*E*)-diphenyl­diazene fragment is 10.92 (13)° and that between the benzotriazole mean plane and the central benzene ring is 80.57 (7)°. In the crystal, π–π stacking [centroid–centroid distances = 3.823 (2) and 3.863 (2) Å] of similar fragments generates mol­ecular layers parallel to (0-12). The crystal packing also features weak C—H⋯N hydrogen bonds involving N atoms of the benzotriazole ring.

## Related literature

For applications of 1-hy­droxy­benzotriazole in organic syntheses, see: König & Geiger (1970 ▶); Miyazawa *et al.* (1984 ▶); Baldini *et al.* (2008 ▶). For the use of 1-hy­droxy­benzotriazole in the preparation of coordination compounds, see: Papaefstathiou *et al.* (2002 ▶).
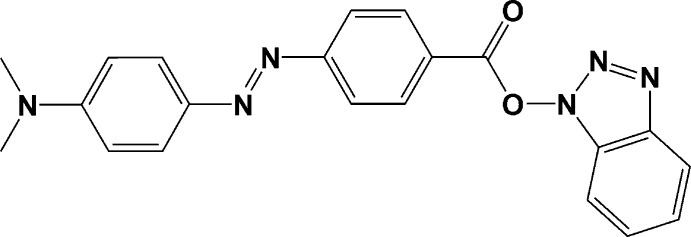


## Experimental

### 

#### Crystal data

C_21_H_18_N_6_O_2_
*M* *_r_* = 386.41
Triclinic, 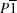

*a* = 6.6362 (8) Å
*b* = 11.384 (3) Å
*c* = 13.022 (3) Å
α = 99.64 (3)°
β = 103.61 (2)°
γ = 92.440 (17)°
*V* = 939.2 (3) Å^3^
*Z* = 2
Mo *K*α radiation
μ = 0.09 mm^−1^
*T* = 293 K
0.3 × 0.2 × 0.2 mm

#### Data collection

Bruker KappaCCD diffractometer
Absorption correction: multi-scan (*SADABS*; Bruker, 2008 ▶) *T*_min_ = 0.697, *T*_max_ = 0.746
18381 measured reflections
4288 independent reflections
2107 reflections with *I* > 2σ(*I*)
*R*_int_ = 0.059

#### Refinement

*R*[*F* ^2^ > 2σ(*F* ^2^)] = 0.062
*wR*(*F* ^2^) = 0.142
*S* = 1.04
4288 reflections
264 parameters
H-atom parameters constrained
Δρ_max_ = 0.20 e Å^−3^
Δρ_min_ = −0.18 e Å^−3^

### 

Data collection: *COLLECT* (Hooft, 1998 ▶); cell refinement: *DIRAX* (Duisenberg, 1992 ▶); data reduction: *EVALCCD* (Duisenberg *et al.*, 2003 ▶); program(s) used to solve structure: *SHELXS97* (Sheldrick, 2008 ▶); program(s) used to refine structure: *SHELXL97* (Sheldrick, 2008 ▶); molecular graphics: *DIAMOND* (Brandenburg, 2005 ▶); software used to prepare material for publication: *WinGX* (Farrugia, 2012 ▶).

## Supplementary Material

Crystal structure: contains datablock(s) global, I. DOI: 10.1107/S1600536813000846/zq2193sup1.cif

Structure factors: contains datablock(s) I. DOI: 10.1107/S1600536813000846/zq2193Isup2.hkl

Supplementary material file. DOI: 10.1107/S1600536813000846/zq2193Isup3.cml

Additional supplementary materials:  crystallographic information; 3D view; checkCIF report

## Figures and Tables

**Table 1:** Hydrogen-bond geometry (Å, °)

*D*—H⋯*A*	*D*—H	H⋯*A*	*D*⋯*A*	*D*—H⋯*A*
C4—H4⋯N5^i^	0.93	2.63	3.415 (3)	142
C23—H23⋯N6^ii^	0.93	2.63	3.560 (3)	176

Symmetry codes: (i) 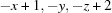; (ii) 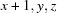.

**Fig. 1.**
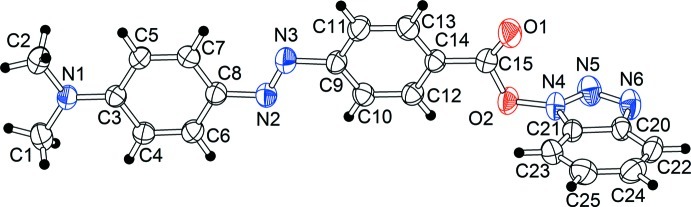
The molecular structure of the title compound. Displacement ellipsoids are drawn at the 50% probability level.

**Fig. 2.**
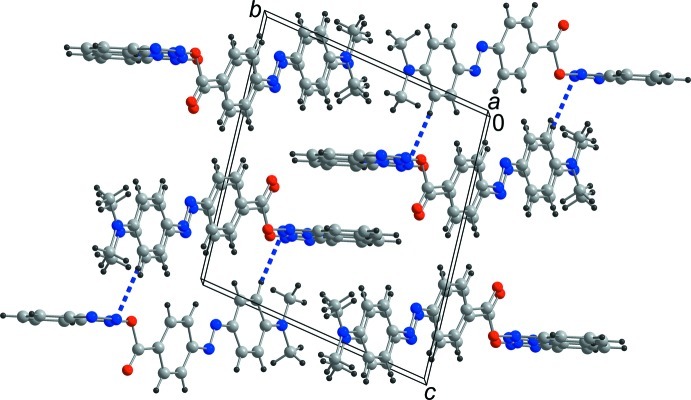
Packing diagram of the title compound viewed along the a–axis. Hydrogen C—H···N bonds are shown as dashed lines.

## supplementary crystallographic information

### Comment

1-Hydroxybenzotriazole is a widely used compound in organic syntheses to decrease the racemization in the carbodiimide peptide coupling (König *et al.*, 1970) and especially in racemization-free condensation of aminoacids and peptidic fragments (Miyazawa *et al.*, 1984). It has also been utilized to form a benzotriazolyl active ester (Baldini *et al.*, 2008). Recently 1-hydroxybenzotriazole was used in the preparation of one-dimensional coordination polymers (Papaefstathiou *et al.*, 2002).

The molecular structure of the title compound is shown in Fig. 1. The diphenyldiazene fragment of the molecule is not planar (its benzene rings form a dihedral angle of 10.92 (13) °) and adopts an *E* conformation about the N2═N3 bond. The benzotriazolyl fragment (tautomer A) is essentially planar with an *r.m.s.* deviation of 0.010 (2) Å and is almost perpendicularly attached to the benzoate ring. The dihedral angles between mean plane of benzotriazolyl and two benzene rings, C3–C8 & C9–C14, are 88.57 (7) ° and 80.57 (7) °, respectively.

In the crystal structure (Fig. 2) π-π stacking of the similar fragments generates molecular layers parallel to (012) [*Cg*1···*Cg*2^i^, 3.823 Å; *Cg*3···*Cg*3^ii^, 3.863 Å; *Cg*1, *Cg*2 and *Cg*3 are the centroids of the C3–C8, C9–C14 and C20–C25 rings, respectively; symmetry codes: (i) 1 + *x*, *y*, *z*; (ii) -3 - *x*, -1 - *y*, -1 - *z*]. Adjacent molecules inside and between the layers are linked additionally by weak C—H···N hydrogen bonds to N-atoms of the benzotriazolyl ring (the shortest H···N distances are 2.63 Å).

### Experimental

The title compound, C_21_H_18_N_6_O_2_, was obtained as a byproduct of a reaction between (*E*)-4-(4-dimethylaminophenylazo)benzoic acid and 2-amino-4-(2-pyridyl)-6-(6-pyridyl)-1,3,5-triazine, which is hardly soluble, under peptidic coupling condition. The condensation reaction has occurred between 1-hydroxybenzotriazole and (*E*)-4-(4-dimethylaminophenylazo)benzoic acid.

### Refinement

Hydrogen atoms were located in a difference electron density map and refined in a riding model (including free rotation for methyl groups), with *U*_iso_(H) = 1.2 (1.5 for methyl groups) times *U*_eq_(C).

### Crystal data

**Table:**

C_21_H_18_N_6_O_2_	*Z* = 2
*M**_r_* = 386.41	*F*(000) = 404
Triclinic, *P*1	*D*_x_ = 1.366 Mg m^−^^3^
Hall symbol: -P 1	Mo *K*α radiation, λ = 0.71073 Å
*a* = 6.6362 (8) Å	Cell parameters from 4814 reflections
*b* = 11.384 (3) Å	θ = 3.7–27.6°
*c* = 13.022 (3) Å	µ = 0.09 mm^−^^1^
α = 99.64 (3)°	*T* = 293 K
β = 103.61 (2)°	Prism, dark-red
γ = 92.440 (17)°	0.3 × 0.2 × 0.2 mm
*V* = 939.2 (3) Å^3^	

### Data collection

**Table:**

Bruker KappaCCD diffractometer	4288 independent reflections
Radiation source: fine-focus sealed tube	2107 reflections with *I* > 2σ(*I*)
Horizontally mounted graphite crystal monochromator	*R*_int_ = 0.059
Detector resolution: 9 pixels mm^-1^	θ_max_ = 27.6°, θ_min_ = 3.7°
combined ω– and φ–scans	*h* = −8→8
Absorption correction: multi-scan (*SADABS*; Bruker, 2008)	*k* = −14→14
*T*_min_ = 0.697, *T*_max_ = 0.746	*l* = −16→16
18381 measured reflections	

### Refinement

**Table:**

Refinement on *F*^2^	Primary atom site location: structure-invariant direct methods
Least-squares matrix: full	Secondary atom site location: difference Fourier map
*R*[*F*^2^ > 2σ(*F*^2^)] = 0.062	Hydrogen site location: difference Fourier map
*wR*(*F*^2^) = 0.142	H-atom parameters constrained
*S* = 1.04	*w* = 1/[σ^2^(*F*_o_^2^) + (0.0421*P*)^2^ + 0.3933*P*] where *P* = (*F*_o_^2^ + 2*F*_c_^2^)/3
4288 reflections	(Δ/σ)_max_ = 0.006
264 parameters	Δρ_max_ = 0.20 e Å^−^^3^
0 restraints	Δρ_min_ = −0.18 e Å^−^^3^

### Special details

**Table:**

Geometry. All s.u.'s (except the s.u. in the dihedral angle between two l.s. planes) are estimated using the full covariance matrix. The cell s.u.'s are taken into account individually in the estimation of s.u.'s in distances, angles and torsion angles; correlations between s.u.'s in cell parameters are only used when they are defined by crystal symmetry. An approximate (isotropic) treatment of cell s.u.'s is used for estimating s.u.'s involving l.s. planes.
Refinement. Refinement of *F*^2^ against ALL reflections. The weighted *R*-factor *wR* and goodness of fit *S* are based on *F*^2^, conventional *R*-factors *R* are based on *F*, with *F* set to zero for negative *F*^2^. The threshold expression of *F*^2^ > 2σ(*F*^2^) is used only for calculating *R*-factors(gt) *etc*. and is not relevant to the choice of reflections for refinement. *R*-factors based on *F*^2^ are statistically about twice as large as those based on *F*, and *R*- factors based on ALL data will be even larger.

### Fractional atomic coordinates and isotropic or equivalent isotropic displacement parameters (Å^2^)

**Table:**

	*x*	*y*	*z*	*U*_iso_*/*U*_eq_	
C1	1.7674 (4)	0.3683 (3)	1.0813 (2)	0.0622 (8)	
H1A	1.8814	0.4273	1.0911	0.093*	
H1B	1.8198	0.2915	1.0858	0.093*	
H1C	1.6942	0.3894	1.1362	0.093*	
C2	1.7041 (4)	0.4217 (3)	0.9009 (2)	0.0616 (8)	
H2A	1.8431	0.4572	0.9338	0.092*	
H2B	1.6156	0.4828	0.8805	0.092*	
H2C	1.7048	0.3639	0.8382	0.092*	
C3	1.4360 (3)	0.3014 (2)	0.94775 (19)	0.0396 (6)	
C4	1.3631 (4)	0.2398 (2)	1.0190 (2)	0.0424 (6)	
H4	1.4470	0.2409	1.0874	0.051*	
C5	1.3021 (4)	0.2962 (2)	0.8449 (2)	0.0481 (7)	
H5	1.3452	0.3359	0.7958	0.058*	
C6	1.1699 (4)	0.1782 (2)	0.9887 (2)	0.0439 (6)	
H6	1.1252	0.1386	1.0373	0.053*	
C7	1.1107 (4)	0.2342 (2)	0.8161 (2)	0.0479 (7)	
H7	1.0259	0.2322	0.7478	0.057*	
C8	1.0396 (3)	0.1736 (2)	0.8874 (2)	0.0421 (6)	
C9	0.5372 (3)	0.0387 (2)	0.7486 (2)	0.0424 (6)	
C10	0.4539 (3)	−0.0039 (2)	0.8252 (2)	0.0418 (6)	
H10	0.5271	0.0114	0.8970	0.050*	
C11	0.4233 (4)	0.0191 (2)	0.6422 (2)	0.0527 (7)	
H11	0.4757	0.0504	0.5912	0.063*	
C12	0.2630 (3)	−0.0688 (2)	0.7946 (2)	0.0421 (6)	
H12	0.2079	−0.0973	0.8459	0.051*	
C13	0.2336 (4)	−0.0462 (2)	0.6120 (2)	0.0516 (7)	
H13	0.1591	−0.0600	0.5404	0.062*	
C14	0.1516 (3)	−0.0921 (2)	0.6875 (2)	0.0405 (6)	
C15	−0.0433 (4)	−0.1686 (2)	0.6479 (2)	0.0459 (6)	
C20	−0.4479 (3)	−0.4709 (2)	0.64582 (19)	0.0422 (6)	
C21	−0.2442 (3)	−0.4259 (2)	0.65840 (19)	0.0396 (6)	
C22	−0.5059 (4)	−0.5924 (2)	0.6047 (2)	0.0531 (7)	
H22	−0.6414	−0.6247	0.5956	0.064*	
C23	−0.0905 (4)	−0.4947 (2)	0.6317 (2)	0.0517 (7)	
H23	0.0451	−0.4627	0.6405	0.062*	
C24	−0.3566 (5)	−0.6615 (2)	0.5784 (2)	0.0587 (8)	
H24	−0.3908	−0.7426	0.5512	0.070*	
C25	−0.1519 (4)	−0.6131 (3)	0.5914 (2)	0.0591 (8)	
H25	−0.0548	−0.6633	0.5720	0.071*	
N1	1.6270 (3)	0.36337 (19)	0.97666 (17)	0.0496 (6)	
N2	0.8454 (3)	0.10799 (17)	0.86454 (18)	0.0453 (5)	
N3	0.7346 (3)	0.10456 (18)	0.77031 (18)	0.0494 (6)	
N4	−0.2534 (3)	−0.30838 (18)	0.69602 (17)	0.0480 (6)	
N5	−0.4415 (3)	−0.2807 (2)	0.70813 (18)	0.0565 (6)	
N6	−0.5633 (3)	−0.3798 (2)	0.67744 (17)	0.0551 (6)	
O1	−0.1551 (3)	−0.19039 (18)	0.55969 (16)	0.0682 (6)	
O2	−0.0874 (2)	−0.22242 (15)	0.73157 (14)	0.0527 (5)	

### Atomic displacement parameters (Å^2^)

**Table:**

	*U*^11^	*U*^22^	*U*^33^	*U*^12^	*U*^13^	*U*^23^
C1	0.0443 (15)	0.076 (2)	0.0604 (19)	−0.0091 (14)	0.0009 (13)	0.0164 (16)
C2	0.0521 (16)	0.0671 (19)	0.067 (2)	−0.0110 (14)	0.0127 (14)	0.0212 (16)
C3	0.0386 (13)	0.0351 (13)	0.0433 (15)	0.0008 (10)	0.0074 (11)	0.0068 (11)
C4	0.0431 (14)	0.0460 (15)	0.0370 (14)	−0.0008 (11)	0.0073 (11)	0.0098 (12)
C5	0.0453 (15)	0.0531 (16)	0.0472 (16)	−0.0056 (12)	0.0082 (12)	0.0198 (13)
C6	0.0437 (14)	0.0479 (15)	0.0430 (16)	−0.0001 (11)	0.0141 (12)	0.0122 (12)
C7	0.0459 (15)	0.0501 (16)	0.0444 (16)	−0.0017 (12)	0.0015 (12)	0.0146 (13)
C8	0.0398 (13)	0.0386 (14)	0.0468 (16)	0.0004 (11)	0.0097 (11)	0.0075 (12)
C9	0.0405 (14)	0.0362 (14)	0.0487 (16)	−0.0021 (11)	0.0094 (12)	0.0068 (12)
C10	0.0419 (14)	0.0373 (14)	0.0406 (15)	0.0002 (11)	0.0039 (11)	0.0014 (12)
C11	0.0540 (16)	0.0581 (17)	0.0452 (17)	−0.0100 (13)	0.0104 (13)	0.0138 (14)
C12	0.0440 (14)	0.0382 (14)	0.0428 (16)	−0.0001 (11)	0.0101 (11)	0.0055 (12)
C13	0.0520 (16)	0.0558 (17)	0.0405 (16)	−0.0077 (13)	0.0010 (12)	0.0084 (13)
C14	0.0396 (13)	0.0334 (13)	0.0452 (16)	−0.0007 (10)	0.0065 (11)	0.0044 (12)
C15	0.0437 (15)	0.0435 (15)	0.0493 (17)	−0.0021 (12)	0.0102 (13)	0.0087 (13)
C20	0.0362 (13)	0.0580 (17)	0.0328 (14)	−0.0067 (12)	0.0087 (10)	0.0113 (12)
C21	0.0362 (13)	0.0451 (15)	0.0359 (14)	−0.0049 (11)	0.0063 (10)	0.0089 (12)
C22	0.0534 (16)	0.0613 (19)	0.0413 (16)	−0.0204 (14)	0.0083 (12)	0.0119 (14)
C23	0.0377 (14)	0.0608 (19)	0.0565 (18)	0.0011 (13)	0.0084 (12)	0.0160 (15)
C24	0.074 (2)	0.0464 (17)	0.0523 (18)	−0.0070 (15)	0.0094 (15)	0.0118 (14)
C25	0.0589 (18)	0.0575 (19)	0.0603 (19)	0.0110 (14)	0.0115 (14)	0.0120 (15)
N1	0.0416 (12)	0.0570 (14)	0.0483 (13)	−0.0109 (10)	0.0056 (10)	0.0157 (11)
N2	0.0389 (11)	0.0432 (12)	0.0513 (14)	−0.0021 (9)	0.0078 (10)	0.0076 (10)
N3	0.0427 (12)	0.0479 (13)	0.0537 (15)	−0.0060 (10)	0.0082 (10)	0.0063 (11)
N4	0.0376 (12)	0.0490 (14)	0.0528 (14)	−0.0105 (10)	0.0106 (10)	0.0007 (11)
N5	0.0465 (13)	0.0686 (16)	0.0533 (15)	0.0000 (12)	0.0184 (11)	0.0007 (12)
N6	0.0410 (12)	0.0723 (16)	0.0512 (14)	−0.0088 (12)	0.0177 (10)	0.0032 (12)
O1	0.0596 (12)	0.0825 (15)	0.0505 (13)	−0.0235 (10)	−0.0060 (10)	0.0137 (11)
O2	0.0494 (10)	0.0538 (11)	0.0474 (11)	−0.0177 (8)	0.0048 (8)	0.0041 (9)

### Geometric parameters (Å, º)

**Table:**

C1—N1	1.449 (3)	C11—C13	1.372 (3)
C1—H1A	0.9600	C11—H11	0.9300
C1—H1B	0.9600	C12—C14	1.391 (3)
C1—H1C	0.9600	C12—H12	0.9300
C2—N1	1.451 (3)	C13—C14	1.391 (3)
C2—H2A	0.9600	C13—H13	0.9300
C2—H2B	0.9600	C14—C15	1.461 (3)
C2—H2C	0.9600	C15—O1	1.191 (3)
C3—N1	1.362 (3)	C15—O2	1.417 (3)
C3—C4	1.410 (3)	C20—N6	1.380 (3)
C3—C5	1.413 (3)	C20—C21	1.388 (3)
C4—C6	1.372 (3)	C20—C22	1.399 (3)
C4—H4	0.9300	C21—N4	1.354 (3)
C5—C7	1.365 (3)	C21—C23	1.385 (3)
C5—H5	0.9300	C22—C24	1.362 (4)
C6—C8	1.389 (3)	C22—H22	0.9300
C6—H6	0.9300	C23—C25	1.369 (4)
C7—C8	1.396 (3)	C23—H23	0.9300
C7—H7	0.9300	C24—C25	1.405 (4)
C8—N2	1.404 (3)	C24—H24	0.9300
C9—C11	1.389 (3)	C25—H25	0.9300
C9—C10	1.392 (3)	N2—N3	1.267 (3)
C9—N3	1.425 (3)	N4—N5	1.339 (3)
C10—C12	1.376 (3)	N4—O2	1.379 (2)
C10—H10	0.9300	N5—N6	1.306 (3)
			
N1—C1—H1A	109.5	C10—C12—H12	119.7
N1—C1—H1B	109.5	C14—C12—H12	119.7
H1A—C1—H1B	109.5	C11—C13—C14	120.6 (2)
N1—C1—H1C	109.5	C11—C13—H13	119.7
H1A—C1—H1C	109.5	C14—C13—H13	119.7
H1B—C1—H1C	109.5	C13—C14—C12	119.0 (2)
N1—C2—H2A	109.5	C13—C14—C15	117.4 (2)
N1—C2—H2B	109.5	C12—C14—C15	123.6 (2)
H2A—C2—H2B	109.5	O1—C15—O2	120.9 (2)
N1—C2—H2C	109.5	O1—C15—C14	129.1 (2)
H2A—C2—H2C	109.5	O2—C15—C14	110.0 (2)
H2B—C2—H2C	109.5	N6—C20—C21	109.7 (2)
N1—C3—C4	121.6 (2)	N6—C20—C22	130.7 (2)
N1—C3—C5	121.3 (2)	C21—C20—C22	119.6 (2)
C4—C3—C5	117.1 (2)	N4—C21—C23	134.8 (2)
C6—C4—C3	120.8 (2)	N4—C21—C20	101.5 (2)
C6—C4—H4	119.6	C23—C21—C20	123.7 (2)
C3—C4—H4	119.6	C24—C22—C20	117.5 (2)
C7—C5—C3	121.2 (2)	C24—C22—H22	121.3
C7—C5—H5	119.4	C20—C22—H22	121.3
C3—C5—H5	119.4	C25—C23—C21	115.4 (2)
C4—C6—C8	121.7 (2)	C25—C23—H23	122.3
C4—C6—H6	119.2	C21—C23—H23	122.3
C8—C6—H6	119.2	C22—C24—C25	121.6 (3)
C5—C7—C8	121.3 (2)	C22—C24—H24	119.2
C5—C7—H7	119.4	C25—C24—H24	119.2
C8—C7—H7	119.4	C23—C25—C24	122.3 (3)
C6—C8—C7	117.9 (2)	C23—C25—H25	118.9
C6—C8—N2	117.1 (2)	C24—C25—H25	118.9
C7—C8—N2	125.0 (2)	C3—N1—C1	122.1 (2)
C11—C9—C10	119.6 (2)	C3—N1—C2	121.0 (2)
C11—C9—N3	115.5 (2)	C1—N1—C2	116.8 (2)
C10—C9—N3	124.9 (2)	N3—N2—C8	114.5 (2)
C12—C10—C9	119.8 (2)	N2—N3—C9	113.9 (2)
C12—C10—H10	120.1	N5—N4—C21	113.82 (19)
C9—C10—H10	120.1	N5—N4—O2	119.6 (2)
C13—C11—C9	120.2 (2)	C21—N4—O2	126.21 (19)
C13—C11—H11	119.9	N6—N5—N4	106.8 (2)
C9—C11—H11	119.9	N5—N6—C20	108.21 (19)
C10—C12—C14	120.7 (2)	N4—O2—C15	112.84 (18)
			
N1—C3—C4—C6	179.7 (2)	C21—C20—C22—C24	−0.1 (4)
C5—C3—C4—C6	−0.2 (3)	N4—C21—C23—C25	−177.4 (3)
N1—C3—C5—C7	−179.9 (2)	C20—C21—C23—C25	−0.2 (4)
C4—C3—C5—C7	0.0 (4)	C20—C22—C24—C25	−0.3 (4)
C3—C4—C6—C8	0.3 (4)	C21—C23—C25—C24	−0.2 (4)
C3—C5—C7—C8	0.1 (4)	C22—C24—C25—C23	0.5 (4)
C4—C6—C8—C7	−0.1 (4)	C4—C3—N1—C1	0.9 (4)
C4—C6—C8—N2	179.9 (2)	C5—C3—N1—C1	−179.2 (2)
C5—C7—C8—C6	−0.1 (4)	C4—C3—N1—C2	177.1 (2)
C5—C7—C8—N2	179.9 (2)	C5—C3—N1—C2	−3.0 (4)
C11—C9—C10—C12	2.3 (4)	C6—C8—N2—N3	−178.6 (2)
N3—C9—C10—C12	−178.6 (2)	C7—C8—N2—N3	1.5 (3)
C10—C9—C11—C13	−2.7 (4)	C8—N2—N3—C9	−179.39 (19)
N3—C9—C11—C13	178.2 (2)	C11—C9—N3—N2	−171.6 (2)
C9—C10—C12—C14	−0.2 (3)	C10—C9—N3—N2	9.4 (3)
C9—C11—C13—C14	0.9 (4)	C23—C21—N4—N5	178.5 (3)
C11—C13—C14—C12	1.2 (4)	C20—C21—N4—N5	0.9 (3)
C11—C13—C14—C15	−175.3 (2)	C23—C21—N4—O2	−8.5 (4)
C10—C12—C14—C13	−1.6 (3)	C20—C21—N4—O2	173.9 (2)
C10—C12—C14—C15	174.7 (2)	C21—N4—N5—N6	−0.7 (3)
C13—C14—C15—O1	−7.1 (4)	O2—N4—N5—N6	−174.2 (2)
C12—C14—C15—O1	176.5 (3)	N4—N5—N6—C20	0.1 (3)
C13—C14—C15—O2	170.8 (2)	C21—C20—N6—N5	0.5 (3)
C12—C14—C15—O2	−5.6 (3)	C22—C20—N6—N5	−178.6 (2)
N6—C20—C21—N4	−0.8 (3)	N5—N4—O2—C15	−99.1 (3)
C22—C20—C21—N4	178.3 (2)	C21—N4—O2—C15	88.3 (3)
N6—C20—C21—C23	−178.8 (2)	O1—C15—O2—N4	6.6 (3)
C22—C20—C21—C23	0.4 (4)	C14—C15—O2—N4	−171.51 (18)
N6—C20—C22—C24	178.8 (3)		

### Hydrogen-bond geometry (Å, º)

**Table:**

*D*—H···*A*	*D*—H	H···*A*	*D*···*A*	*D*—H···*A*
C4—H4···N5^i^	0.93	2.63	3.415 (3)	142
C23—H23···N6^ii^	0.93	2.63	3.560 (3)	176

Symmetry codes: (i) −*x*+1, −*y*, −*z*+2; (ii) *x*+1, *y*, *z*.

## References

[bb1] Baldini, L., Sansone, F., Faimani, G., Massera, C., Casnati, A. & Ungaro, R. (2008). *Eur. J. Org. Chem.* **5**, 869–886.

[bb2] Brandenburg, K. (2005). *DIAMOND.* Crystal Impact GbR, Bonn, Germany.

[bb3] Bruker (2008). *SADABS* Bruker AXS Inc., Madison, Wisconsin, USA.

[bb4] Duisenberg, A. J. M. (1992). *J. Appl. Cryst.* **25**, 92–96.

[bb5] Duisenberg, A. J. M., Kroon-Batenburg, L. M. J. & Schreurs, A. M. M. (2003). *J. Appl. Cryst.* **36**, 220–229.

[bb6] Farrugia, L. J. (2012). *J. Appl. Cryst.* **45**, 849–854.

[bb7] Hooft, R. W. W. (1998). *COLLECT* Nonius BV, Delft, The Netherlands.

[bb8] König, W. & Geiger, R. (1970). *Chem. Ber.* **103**, 788–798. 10.1002/cber.19701030319 5436656

[bb9] Miyazawa, T., Otomatsu, T., Yamada, T. & Kuwata, S. (1984). *Tetrahedron Lett.* **25**, 771–772.

[bb10] Papaefstathiou, G. S., Vicente, R., Raptopoulou, C. P., Terzis, A., Escuer, A. & Perlepes, S. P. (2002). *Eur. J. Inorg. Chem.* **9**, 2488–2493.

[bb11] Sheldrick, G. M. (2008). *Acta Cryst.* A**64**, 112–122. 10.1107/S0108767307043930 18156677

